# High-fat diet induces senescence in ADSCs via CDK4 ubiquitination-mediated cell cycle disruption, contributing to impaired glucose tolerance

**DOI:** 10.1016/j.molmet.2025.102293

**Published:** 2025-11-29

**Authors:** Zheng Ge, Zitian Liu, Shuohui Dong, Xiang Zhao, Guangwei Yang, Ao Yu, Wei Guo, Xiang Zhang, Qunzheng Wu, Kexin Wang

**Affiliations:** 1 Cheeloo College of Medicine, Shandong University, Jinan, Shandong, China; 2 Department of General Surgery, Qilu Hospital of Shandong University, Jinan, Shandong, China

**Keywords:** Stem cell, Ubiquitination, Cell cycle, Cellular senescence, Adipocyte, Glucose tolerance

## Abstract

High-fat diet (HFD) promotes adipose tissue senescence, which in turn disrupts insulin-mediated glycemic homeostasis. The underlying mechanisms remain unclear. Through clinical survey data, animal models, and primary adipose-derived mesenchymal stem cells (ADSC), we investigated how dietary patterns influence adipocyte senescence. We found that elevated fatty acid levels enhance the interaction between the E3 ubiquitin ligase TRIP12 and Cyclin-dependent kinase 4 (CDK4) in ADSCs, triggering CDK4 ubiquitination and degradation. As a process associated with this disruption in cell cycle progression, cellular senescence may represent a key outcome. Consequently, senescent ADSC-derived mature adipocytes (ADSC-MA) exhibit impaired insulin-stimulated GLUT4 membrane translocation and reduced glucose uptake. In contrast, within an HFD setting, dietary fiber supplementation is associated with the reversal of cellular senescence. The gut microbiota–short-chain fatty acids (SCFAs) axis may be involved in the restoration of cell cycle progression and the amelioration of ADSC senescence, correlating with a partial recovery of glucose uptake capacity in ADSC-MAs. Our study highlights potential strategies to reverse cellular senescence and identifies promising therapeutic targets for impaired glucose tolerance.

## Highlights

• HFD triggers ADSC senescence independently of obesity, a phenomenon that may be linked to elevated levels of NEFAs.
• Senescent ADSCs differentiate into senescent adipocytes with disruption of GLUT4 membrane translocation.
• TRIP12-mediated CDK4 ubiquitination bridges lipotoxicity to ADSC senescence through cell cycle arrest.
• In a high-fat diet setting, dietary fiber reverses senescence, hinting the involvement of a gut microbiota-SCFAs-CDK4 axis.

## Graphical abstract

Our study shows that an HFD increases circulating NEFAs. In this context, we demonstrate that NEFAs promote the binding of TRIP12 to CDK4 in ADSCs, leading to ubiquitination and subsequent degradation of CDK4. The loss of CDK4 disrupts the cell cycle and induces cellular senescence in ADSCs. Senescent ADSCs differentiate into dysfunctional MAs, which exhibit impaired insulin sensitivity and defective insulin-mediated GLUT4 membrane translocation. Consequently, glucose uptake in MAs is significantly diminished. It is thus plausible that this reduction contributes to the manifestation of impaired glucose tolerance at the systemic level. Dietary fiber supplementation alters the gut microbiota composition, increasing SCFAs production. These SCFAs act directly on ADSCs to restore CDK4 protein levels, rescue cell cycle progression, and reverse cellular senescence. This functional recovery of ADSCs suggests that targeting CDK4 restoration could represent a novel therapeutic strategy for HFD-related metabolic disorders.

**Figure:**
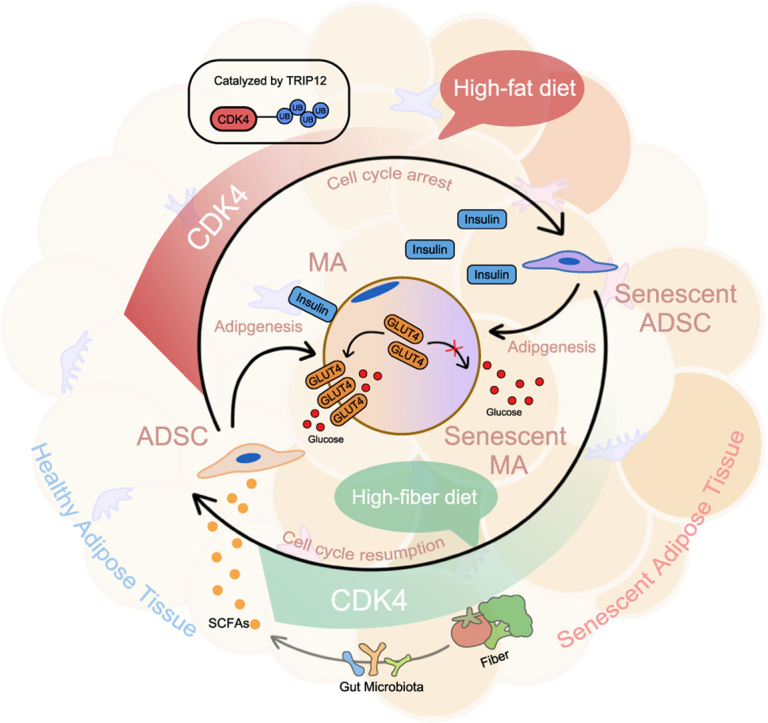

## 1 Introduction

Impaired glucose tolerance is often caused by insulin resistance, which is closely associated with obesity [1,2]. High fat intake is a direct cause of obesity. It is well-established that obesity accelerates cellular senescence in tissues and organs, though the underlying mechanisms remain incompletely understood [3,4]. Proposed mechanisms include inflammation-related pathway activation and oxidative stress [[4], [5], [6]]. However, the direct contribution of an HFD itself has received insufficient attention. Cellular senescence is defined by an irreversible cell cycle arrest, which contributes to functional decline in tissues [7,8]. Adipose tissue plays a vital role in systemic glucose homeostasis, and its senescence is directly associated with metabolic impairment [9,10]. Senescent adipocytes display reduced expression and disrupted function of GLUT4, the major insulin-responsive glucose transporter, ultimately leading to diminished glucose uptake and glucose intolerance [11].

Adipocytes originate from ADSCs, which are capable of robust proliferation due to their active cell cycle and serve as an adipocyte reservoir, indirectly contributing to systemic glucose homeostasis [12]. CDKs are key regulators of cell cycle progression. Among them, CDK4 complexed with cyclin D drives the G1/S transition through RB phosphorylation and subsequent transcription factor release [13,14]. Although CDK4 inhibition induces cellular senescence in breast cancer, its function in subcutaneous adipose tissue remains unknown [15]. The ubiquitin-proteasome system further regulates cell cycle and cellular senescence via targeted degradation of key regulatory proteins [16,17].

A high-fiber diet attenuates cellular senescence and improves glucose tolerance, acting primarily through gut microbiota modulation despite being indigestible [[18], [19], [20]]. Its effects are mediated through the gut microbiota–SCFAs axis, a pathway previously associated with metabolic and developmental regulation [21,22]. However, the potential of this mechanism to reverse cell cycle arrest and counteract cellular senescence in adipose tissue remains unexplored.

Therefore, this study utilizes ADSCs from subcutaneous tissue to explore how divergent dietary habits influence cellular senescence and glucose tolerance, and to investigate the connection between these phenomena.

## 2 Methods

### 2.1 Human study

This study enrolled patients with early-stage colorectal cancer from Qilu Hospital of Shandong University. Exclusion criteria included prior neoadjuvant therapy, type 2 diabetes, or other metabolic disorders. Participant characteristics are detailed in the Supplementary Materials (Tables S1, S2, S3). Dietary fat intake was evaluated using a culturally adapted questionnaire based on the instrument by H. Francis et al. (Table S4) [23]. Fasting blood glucose and blood samples were collected from all participants. Subcutaneous adipose tissue was obtained from 16 individuals undergoing Miles surgery. Participants were stratified into lower fat (score <40) or higher fat (score >50) intake groups, with clinical characteristics closely matched. The study protocol was approved by the Ethics Committee of Qilu Hospital of Shandong University (KYLL-202408-043-1), is compliant with the most recent version of the Helsinki Declaration, and was registered with the Chinese Clinical Trial Registry (ChiCTR2500096934).

### 2.2 Animal study

Male Wistar rats (6–8 weeks old) from Vital River Laboratory Animal Technology Co., Ltd. were housed under a 12-hour light/dark cycle at 24–26 °C and 50–70% humidity. After acclimatization, one batch of rats was fed for 16 weeks with either a control diet (10% wt/wt fat, XTCON50H) or high-fat diet (45% wt/wt fat, XTHF45); another batch received the same initial diets for 16 weeks followed by an additional 16 weeks with one of four dietary treatments: continued control diet, continued high-fat diet, control diet with inulin (37 g/1000 kcal), or high-fat diet with inulin (Figure 2, Figure 6A) [22]. Naturally aged rats were maintained on the control diet for over 24 months [24]. All diets were obtained from Xietong Pharmaceutical Bio-Engineering Co., Ltd. Body weight and fasting glucose were monitored weekly. Body fat composition was analyzed using minispec LF90II (Bruker) at the end of respective experimental periods. Rats were euthanized for serum and subcutaneous inguinal adipose tissue collection. OGTT was performed using 20% (wt/wt) glucose (5 mL/kg) [25]. The sample size (5–6 rats per group) was determined based on previous studies [26]. Randomization was performed using a random number table. Blinding was unnecessary as the intervention and measurements are unlikely to introduce bias. All procedures were approved by the Ethics Committee of Qilu Hospital of Shandong University (DWLL-2024-097).

### 2.3 Primary cell isolation and culture

MA were isolated by mincing adipose tissue and digesting it in a digestion buffer prepared according to the method of Qian Li et al. [27]. at 37 °C with shaking for 60 min. The digest was filtered through a 300-μm mesh (PluriSelect, 43-50300-03) and centrifuged. The adipocyte layer was collected and washed at least three times with Dulbecco's PBS, then cultured floating in Adipocyte Medium (Sciencell, 7201).

ADSCs were isolated following the method of Aina Lluch et al. [28]. The initial steps were identical to MA isolation. After filtration, the sample was centrifuged and the pellet was cultured in DMEM/F12 (Gibco,11320033) supplemented with 20% (vol/vol) FBS (Gibco, 10099141) and 1% (vol/vol) antibiotic-antimycotic solution (Solarbio, P7630) for 24 h. Non-adherent cells were removed by Dulbecco's PBS washing, and the medium was refreshed. Cells after two passages were used for experiments.

For adipogenic differentiation, ADSCs were induced following established protocols using a standard cocktail [29]. Briefly, cells were treated with differentiation medium for 4 days. The medium consisted of DMEM/F12 supplemented with 20% FBS, 1% antibiotic-antimycotic solution, 5 μg/ml insulin (Sigma I3536), 1 μM dexamethasone (Sigma D1756), 0.5 μM rosiglitazone (Sigma R2408), and 500 μM IBMX (Sigma I5879). Thereafter, the culture was continued without dexamethasone, rosiglitazone, and IBMX. Cells were then cultured for 3 days for flow cytometry or 6 days for other assays. Only cells with microscopically confirmed lipid droplet formation were utilized in subsequent experiments.

For lipotoxic stimulation, cells were treated with high-fat medium containing 500 μmol/l sodium palmitate (Sigma–Aldrich, p0500) for 12 h.

The SCFAs cocktail was formulated to reflect serum levels observed in rats, containing 0.3 μg/mL sodium propionate (Aladdin, S100121), 1 μg/mL sodium butyrate (Aladdin, S102954), 0.06 μg/mL sodium valerate (Aladdin, S194180), 0.06 μg/mL isovaleric acid (Aladdin, I108281), and 0.2 μg/mL sodium hexanoate (Aladdin, S161313), dissolved directly in the culture medium and applied either alone or in combination with high-fat medium.

For CDK4 inhibition, cells were treated with 5 μM Palbociclib (Selleck, S1116) for 24 h before collection.

### 2.4 Oil red O staining

Cells were stained after adipogenic differentiation using an Oil Red O staining kit (Solarbio, G1262) according to the manufacturer's protocol. After hematoxylin counterstaining, cells were observed under distilled water.

### 2.5 Histological section preparation

Paraffin sections of fixed adipose tissue were dehydrated using a HistoCore PEARL automated processor (Leica), embedded, and sectioned at 5 μm. For frozen sections, fresh adipose tissue was embedded in OCT compound and sectioned at 20 μm using a CM1950 cryostat (Leica).

### 2.6 Immunofluorescence and immunohistochemistry

Cells were incubated overnight with primary antibodies after fixation and blocking. Following incubation with a fluorophore-conjugated secondary antibody for 2 h and DAPI staining, imaging was performed using an Axio Vert.A1 fluorescence microscope (Zeiss).

For tissue immunofluorescence, paraffin sections underwent deparaffinization, gradient alcohol treatment, and antigen retrieval before following the cellular immunofluorescence protocol.

For immunohistochemistry, frozen sections were fixed and antigen-retrieved, then stained using an immunohistochemistry kit (ZSGB-BIO, PV9000).

Antibodies used are described in the Supplementary Materials (Table S5).

### 2.7 RNA-seq analysis

Total RNA was extracted using Trizol reagent. Poly(A) + RNA was enriched, fragmented, and reverse-transcribed into cDNA. Sequencing libraries were prepared and paired-end sequencing was performed on an Illumina Novaseq™ X Plus platform. DESeq2 was used to identify DEGs, followed by enrichment analyses using GO and KEGG. The raw data are available in the NCBI database under accession numbers PRJNA1335807 and PRJNA1335831.

### 2.8 SA-β-gal staining

SA-β-Gal staining was performed using a commercial kit (Solarbio, G1580) according to the manufacturer's protocol. Cultured cells were stained for 6 h, with ADSCs counterstained using Nuclear Fast Red (Solarbio, G1321). Frozen sections were stained for 24 h and also counterstained with Nuclear Fast Red.

### 2.9 Protein sample preparation

Total cellular protein was extracted using lysis buffer containing protease inhibitor. After centrifugation, the supernatant was boiled in loading buffer at 95 °C for 5 min.

For adipocyte membrane protein, cells were stimulated with 5 μg/mL insulin for 1 h prior to extraction using a membrane protein extraction kit (Beyotime, P0033). Samples were incubated with loading buffer at 37 °C for 30 min.

For adipose tissue protein, extracts were prepared using a dedicated adipose tissue protein extraction kit (Solarbio, EX1130).

### 2.10 WB

Gels were prepared using a commercial kit (Epizyme, PG212/PG214). Following electrophoresis, proteins were transferred to 0.45 μm PVDF membranes (Millipore, IPVH00010). After blocking, membranes were incubated with primary antibodies at 4 °C overnight. Membranes were then incubated with HRP-conjugated secondary antibodies for 2 h, and signals were detected using ECL reagent (Proteintech, PK10002). Antibodies used are described in the Supplementary Materials (Table S5).

### 2.11 Reverse transcription-qPCR

Total RNA was extracted using a commercial kit (Goonie, 400-105), reverse-transcribed with ReverTra Ace qPCR RT Master Mix (TOYOBO, FSQ-301), and analyzed by qPCR on a LightCycler 480 II (Roche) using SYBR Green Master Mix (TOYOBO, QPK-201). Gene expression was calculated using the 2–ΔΔCt method normalized to cyclophilin A [28]. Primer sequences are provided in the Supplementary Information (Table S6).

### 2.12 Insulin measurement by ELISA

Serum insulin levels were quantified using the Human Insulin Valukine™ ELISA Kit (Novus, VAL146) and Rat Insulin ELISA Kit (Reed Biotech, RE3153R) according to manufacturer protocols. Absorbance was measured at 450 nm, with concentrations determined from standard curves.

### 2.13 NEFA measurement

Serum NEFA levels were quantified using a commercial assay kit (Nanjing Jiancheng Bioengineering Institute, A042-2-1) according to the manufacturer's instructions. Absorbance was measured at 546 nm.

### 2.14 Glucose uptake assay

Adipocytes were starved for 4 h in sugar-free DMEM/F12 (Procell, PM150322) containing 20% FBS, then incubated with 100 μM 2-NBDG (Beyotime, ST2078) and 5 μg/mL insulin for 6 h. Fluorescence intensity was measured via FITC channel using a FACS Celesta flow cytometer (BD Bioscience) and analyzed with FlowJo V10.8.1. Cells without 2-NBDG treatment served as NC.

### 2.15 EdU proliferation analyses

Cultured cells were treated with 10 μM EdU for 6 h and detected using a commercial proliferation kit (CellorLab, CX002). Imaging was performed with an Axio Vert.A1 fluorescence microscope.

For adipose tissue analysis, EdU was administered by intraperitoneal injection at 200 mg per kg body weight every 24 h for a total of three injections. Animals were euthanized 24 h after the final injection, and paraffin-embedded adipose sections were stained using the same protocol for imaging.

### 2.16 Cell transfection

ADSCs were transfected with target-specific or non-targeting siRNA (siCtrl) using Lipomaster 3000 (Vazyme, TL301) to silence CDK4 and TRIP12. For CDK4 overexpression, ADSCs were transfected with a CDK4 plasmid or empty vector using Lipomaster 3000, following Shuang Song et al.'s method [30]. Protein was extracted 72 h after transfection for WB efficiency validation (Figs. S3f, g, i). All sequences are provided in the Supplementary Materials (Tables S7 and S8).

### 2.17 Protein stability analysis

Based on the method of Jizhen Li et al. [31]. with modifications, cells were treated at approximately 80% confluence. 50 μg/ml CHX (Aladdin,C112766) was added to the medium to inhibit protein synthesis. Protein was extracted after 2 h of treatment for subsequent analysis.

### 2.18 Co-IP

Co-IP was performed using an anti-CDK4 antibody (Abcam, ab226474) and a commercial Co-IP kit (Thermo, 26149) according to the manufacturer's protocol. Precipitated samples were used for subsequent WB or protein identification.

### 2.19 MS-based protein identification

Protein samples were digested with trypsin after reduction and alkylation, then desalted using C18 columns. Peptides were separated by nano-liquid chromatography with a gradient of 0.1% (vol/vol) formic acid in water and acetonitrile, and analyzed by Q-Exactive mass spectrometry (Thermo). Data were processed using Proteome Discoverer 2.1 for identification.

### 2.20 16S rRNA microbiome sequencing

Fecal DNA was extracted, quantified, and the 16S rRNA V3–V4 region was amplified using primers 341F/805R. Purified PCR products were sequenced on an Illumina NovaSeq 6000 platform (PE250). Bioinformatic processing included quality control, chimera removal, ASV clustering, and taxonomic annotation against the SILVA database. The raw data are available in the NCBI database under accession numbers PRJNA1336216.

### 2.21 Serum SCFAs assay

Serum samples were protein-precipitated with pre-chilled 80% (vol/vol) methanol, vortexed, and centrifuged. The supernatant was derivatized with EDC/3-NPH and analyzed by LC-MS/MS using an Agilent Poroshell column with MRM detection in ESI− mode. Quantification was performed using internal standard calibration curves.

### 2.22 Statistical analysis

Data were analyzed using GraphPad Prism 10.1.2. Statistical methods included Spearman's correlation, linear regression, t-tests, ANOVA, Kruskal–Wallis test, or Bonferroni's test as appropriate. Error bars represent mean ± SEM to emphasize estimation precision, or mean ± SD to show data dispersion. Significance levels: ∗*P* < 0.05, ∗∗*P* < 0.01, ∗∗∗*P* < 0.001, ns nonsignificant; specific comparisons are denoted with symbols as detailed in figure legends.

## 3 Result

### 3.1 High fat intake links to glucose intolerance and adipose tissue senescence

**Figure 1:**
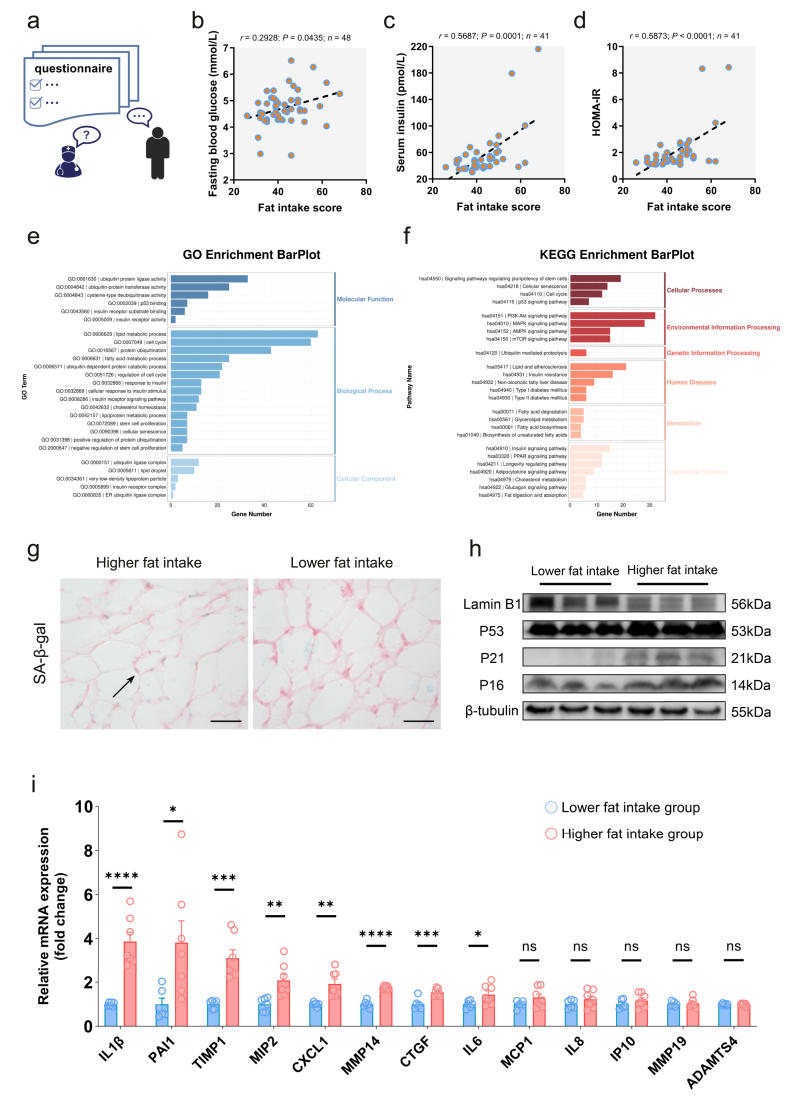
**High fat intake links to glucose intolerance and adipose tissue senescencea** Dietary preference questionnaire survey. Created with BioGDP.com [51] **b**-**d** The correlation of fat intake score with **b** fasting blood glucose, **c** fasting serum insulin, and **d** HOMA-IR. Each dot represents one individual. Two-tailed Spearman's correlation analysis was performed. Trend line by linear regression. **e** GO and **f** KEGG enrichment analysis comparing the higher fat intake group versus the lower fat intake group. **g** SA-β-gal staining in human adipose tissue. Scale bar, 50 μm **h** WB of senescence markers (Three independent samples per group). **i** RT-qPCR analysis of SASP in lower fat intake (n = 6) and higher fat intake (n = 7) groups. Results are presented as mean ± SEM. Statistical significance was assessed by two-tailed *t*-tests. ns non-significant; ∗∗∗∗*P* < 0.0001; ∗∗∗*P* < 0.001; ∗∗*P* < 0.01; ∗*P* < 0.05.

To investigate the impact of high fat intake on glucose tolerance,we assessed habitual fat intake of human volunteers (Figure 1A). Higher fat consumption was significantly associated with elevated fasting blood glucose and increased insulin resistance (Figure 1B–D). The positive correlation between fat intake and fasting insulin levels (Fig. 1C) may represent a compensatory response to maintain glycemic stability, a phenomenon that mirrors the hyperinsulinemia observed in obesity.

To identify key pathways through which high fat intake impairs glucose tolerance, we conducted RNA-seq on subcutaneous adipose tissue from groups with higher and lower fat intake. GO and KEGG analysis of DEGs revealed significant enrichment in cellular senescence-related terms, along with lipid metabolism-associated terms (Figure 1E,F). SA-β-gal activity was increased in subjects with high fat intake (Fig. 1G). Protein levels of senescence markers and SASP mRNA expression also exhibited senescent patterns in these individuals (Figure 1H,I). These results strongly support a close association between high fat intake and adipose tissue senescence.

Intriguingly, we unexpectedly observed prominent SA-β-Gal staining in the SVF of adipose tissue from high-fat intake subjects (Fig. S1). As the SVF contains heterogeneous cell types, the specific cellular origin of this signal remains unclear. Nonetheless, these findings indicate that high fat intake-associated cellular senescence is not restricted to MAs.

### 3.2 HFD triggers adipose tissue senescence independently of obesity, concomitant with metabolic dysregulation

**Figure 2:**
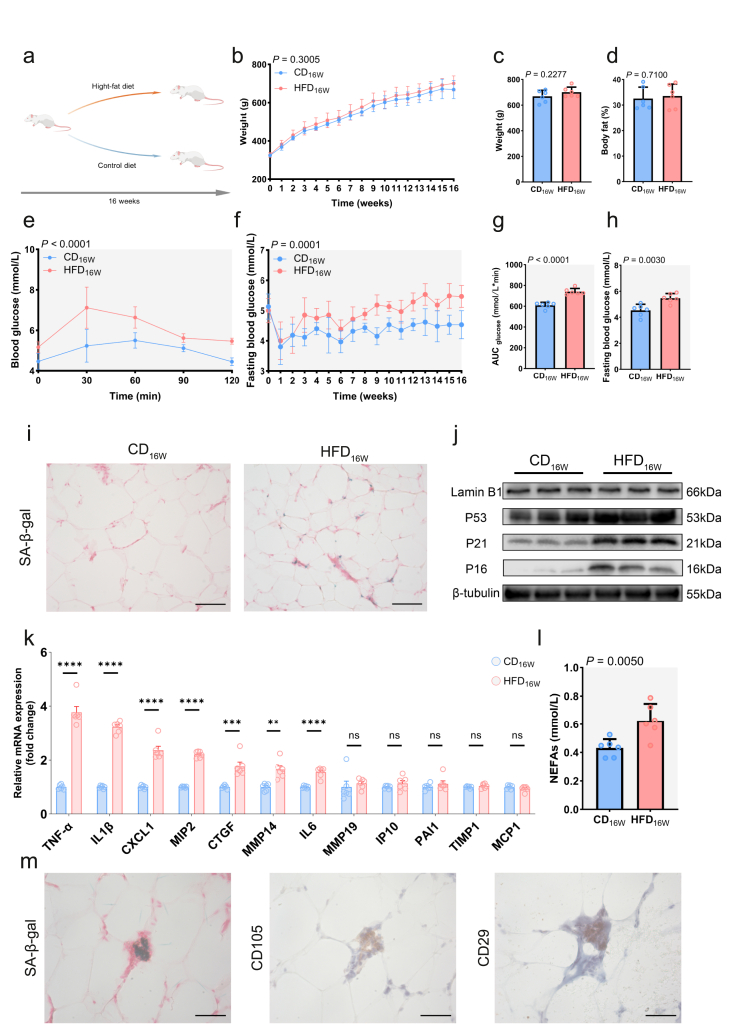
**HFD triggers adipose tissue senescence independently of obesity, concomitant with metabolic dysregulationa** Schematic diagram of the rat feeding protocol. Created with BioGDP.com [51] **b** Body weight changes in rats during the experimental period. **c** Body weight and **d** body fat of rats at week 16. **e** OGTT curves at week 16. **f** Fasting blood glucose levels during the experimental period. **g** AUC analysis of OGTT. **h** Fasting glucose comparison at week 16. Adipose tissue senescence analyses included **i** SA-β-gal staining (Scale bar,100um), **j** senescence markers detection by WB (three separate biological replicates), and **k** SASP mRNA level measurement by RT-qPCR. **l** Serum NEFA levels. **m** Representative adipose tissue section from a HFD_16W_ rat showing co-localization of SA-β-gal activity with ADSC markers CD29 and CD105 (Scale bar,50um). For panels **b**–**h** and **l**, data in line graphs were analyzed by two-way ANOVA, while between-group comparisons in bar graphs were evaluated using two-tailed Student's t-tests, Gray shading indicates statistical significance (*P* < 0.05). All data are presented as mean ± SD. For panel **k**, data were analyzed by two-tailed Student's t-test and are presented as mean ± SEM. Statistical significance was assessed by two-tailed t-tests. ns non-significant; ∗∗∗∗P < 0.0001; ∗∗∗P < 0.001; ∗∗P < 0.01; ∗P < 0.05. All statistical analyses were performed with n = 6 biologically independent replicates.

To further explore the connection between an HFD and adipose tissue senescence, we established CD and HFD rat models (Fig. 2A). After 16 weeks, both CD-fed rats (CD_16W_) and HFD-fed rats (HFD_16W_) showed comparable body weight and fat mass (Figure 2B–D), yet HFD_16W_ developed significant glucose intolerance (Figure 2E–H), indicating impaired glucose tolerance develops independently of obesity. The HFD_16W_ group also exhibited hyperinsulinemia and elevated insulin resistance (Fig. S2a and b), which directly explains the impaired glucose tolerance in these rats.

Adipocytes and adipose tissue senescence are often considered key contributors to reduced insulin sensitivity [9]. We observed multiple features of senescence in the adipose tissue of HFD_16W_ (Figure 2I–K). While obesity is known to promote adipocyte senescence, our rats—after 16 weeks of HFD feeding—did not meet criteria for obesity (Figure 2B–D), yet still exhibited adipose tissue senescence, indicating that HFD induces senescence independently of obesity. Despite no onset of obesity, HFD-fed rats showed higher circulating NEFAs (Fig. 2L), leading us to hypothesize that NEFAs might directly induce cellular senescence.

In adipose tissue, MAs are the main cells in maintaining glucose homeostasis. We isolated primary MAs from rats and stimulated them with PA, but did not observe any SA-β-Gal-positive areas (Fig. S3a). Based on our previous observation of SA-β-Gal-positive areas in the SVF of human tissue (Fig. S1), we turned our attention to ADSCs—the precursor cells of MAs. In the SVF of HFD-fed rat adipose tissue, we clearly detected SA-β-Gal positive regions that also exhibited positive expression of ADSC markers (Fig. 2M), implying that ADSCs play a critical mediating role in HFD-induced adipose tissue senescence.

### 3.3 NEFA-driven ADSC senescence is associated with GLUT4 membrane translocation impairment in adipocytes, along with a diminished capacity for glucose uptake

**Figure 3:**
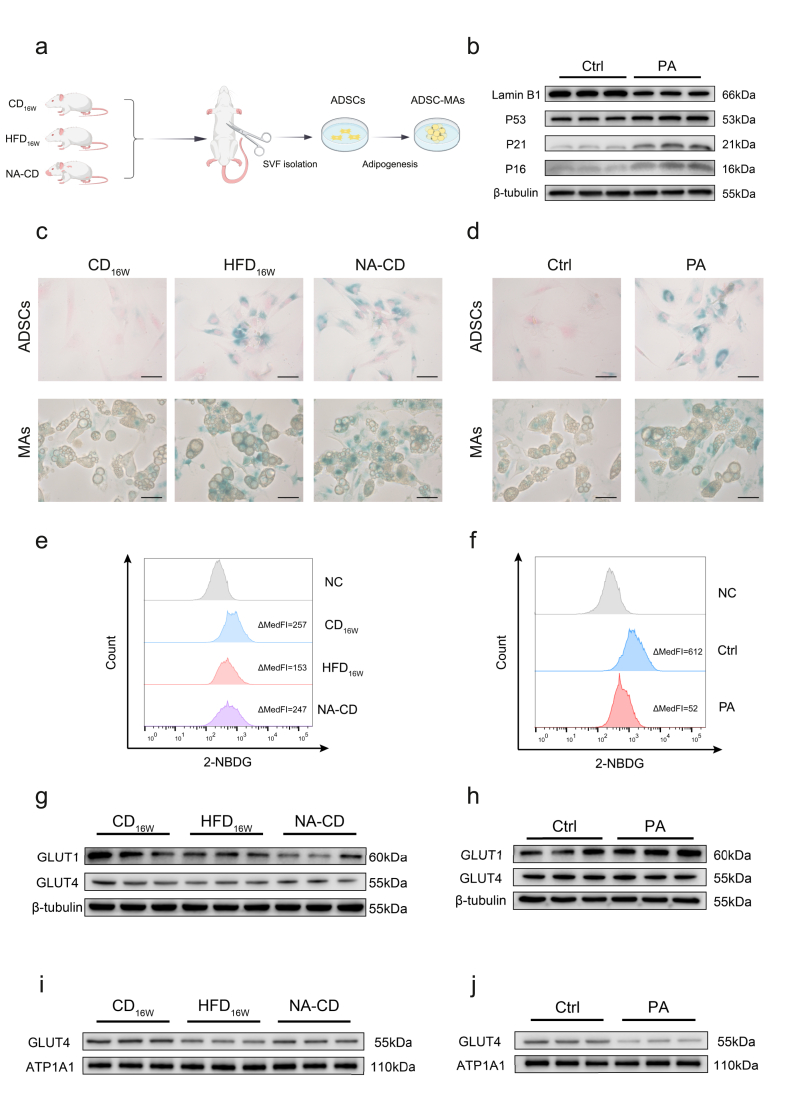
**NEFA-driven ADSC senescence is associated with GLUT4 membrane translocation impairment in adipocytes, along with a diminished capacity for glucose uptakea** Schematic overview of experimental plan. Created with BioGDP.com [51] **b** Representative WB images showing senescence markers in primary ADSCs from the CD_16W_ group treated with PA or control (Ctrl). **c**, **d** SA-β-gal staining of ADSCs and corresponding ADSC-MAs demonstrating cellular senescence **c** in different rat groups and **d** under different treatment of CD_16W_ group-derived cells. Scale bar represents 50um. **e**, **f** Representative flow cytometric analysis of 2-NBDG uptake in ADSC-MAs. ΔMedFI as a measure of glucose uptake capacity. **e** from different rat groups and **f** under different treatment of CD_16W_ group-derived cells. **g**, **h** Total glucose transporters in primary ADSC-MAs from **g** different rat groups and **h** CD_16W_ group-derived cells with varied treatment; **i**, **j** Membrane GLUT4 in the corresponding ADSC-MAs from **i** and **j**. In all WB images, three biologically independent replicates are shown.

ADSCs were isolated from CD-fed, HFD-fed, and naturally aged rats (NA-CD) and subjected to adipogenic differentiation (Figure 3A). Positive expression of ADSC markers and successful adipogenic differentiation confirmed the efficiency of our isolation protocol (Fig. S3b–d). Both HFD_16W_ and NA-CD groups showed marked senescence in both ADSCs and ADSC-MAs (Fig. 3C). To model elevated circulating NEFAs, ADSCs from CD-fed rats were treated with PA in vitro. This treatment in ADSCs triggered senescence in both ADSCs and the resulting differentiated adipocytes (hereafter designated as PA-ADSC-MAs) (Figure 3B,D). In the absence of insulin, none of the PA-ADSC-MAs exhibited significant glucose uptake (Fig. S3e). However, under insulin stimulation, ADSC-MAs from the NA-CD and HFD_16W_ group demonstrated impaired glucose uptake (Fig. 3E), establishing a link between adipocyte senescence and reduced insulin sensitivity. Similarly, PA-ADSC-MAs also showed reduced glucose uptake in response to insulin (Fig. 3F), demonstrating that elevated fatty acids directly promote cellular senescence, which is associated with impaired glucose uptake.

We examined total protein levels of GLUT4 in ADSC-MAs from different groups and found significant downregulation only in the NA-CD group, but not in the HFD_16W_ group (Fig. 3G). GLUT1 expression varied considerably within groups. PA-ADSC-MAs showed unchanged GLUT4 but upregulated GLUT1 (Fig. 3H). Although this seemed inconsistent with the observed reduction in glucose uptake, previous studies suggest that GLUT4 membrane translocation—rather than its total expression—plays a more critical role [32]. We therefore isolated membrane proteins after insulin stimulation. A reduction in membrane GLUT4 was observed in ADSC-MAs from both in vivo (NA-CD and HFD_16W_ groups) and in vitro (PA-ADSC-MAs) models (Figure 3I,J). The upregulation of GLUT1 may represent a compensatory response. These data indicate that ADSC senescence triggered by HFD in vivo or by NEFAs in vitro coincides with a deficit in insulin-stimulated glucose uptake in ADSC-MAs, which is coupled with impaired GLUT4 membrane translocation.

### 3.4 The loss of CDK4 bridges lipotoxicity to ADSC senescence through cell cycle arrest

**Figure 4:**
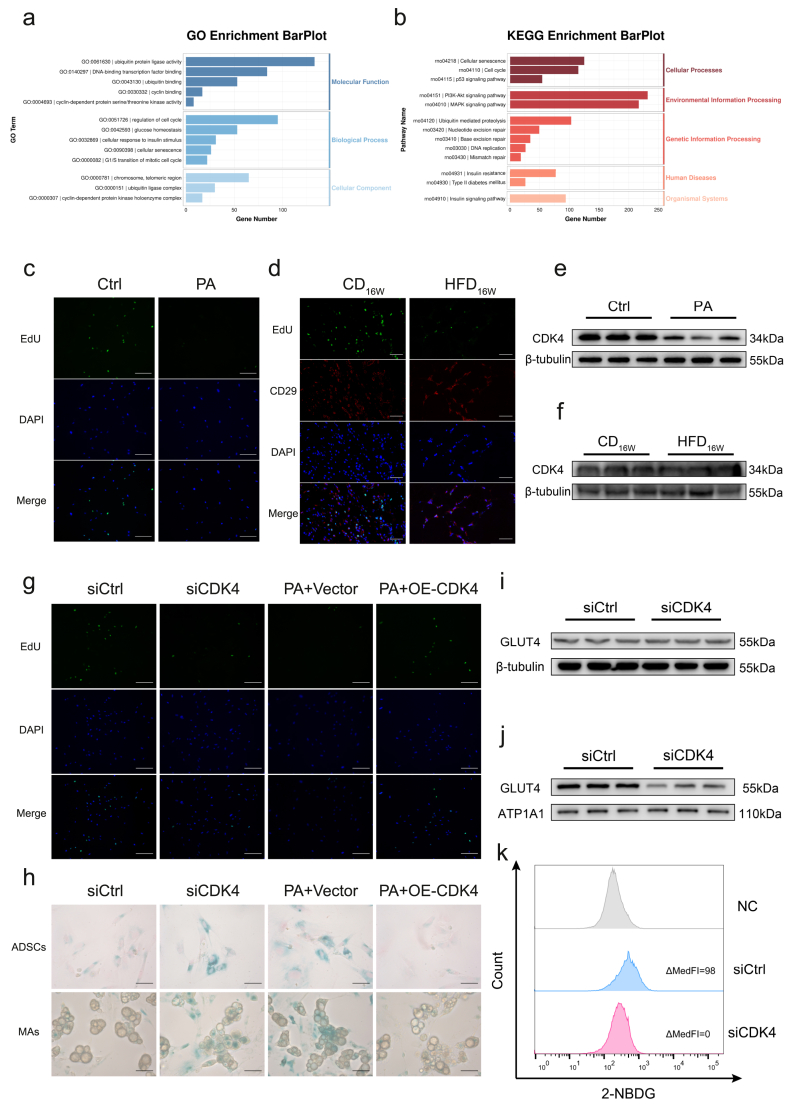
**The loss of CDK4 bridges lipotoxicity to ADSC senescence through cell cycle arresta** GO and **b** KEGG enrichment analysis comparing the ADSC treated with PA versus Ctrl. **c**, **d** EdU staining in **c** cultured ADSCs and **d** rat adipose tissue (with CD29 as ADSC marker). **e**, **f** WB images of CDK4 expression in **e** ADSCs and **f** adipose tissues. **g** EdU staining of ADSCs under indicated treatments. **h** SA-β-gal staining of ADSCs/ADSC-MAs under indicated treatments. **i**, **j** WB images of **i** total GLUT4 and **j** membrane GLUT4 in ADSC-MAs. **k** Flow cytometric analysis of 2-NBDG uptake in ADSC-MAs. For **c** and **g**, Scale bar represents 200um; for **d** and **h**, Scale bar represents 50um. In all WB images, three biologically independent replicates are shown.

Transcriptomic analysis revealed significant enrichment of DEGs in cell cycle-related pathways, in addition to metabolic and senescence pathways, by KEGG and GO analysis (Figure 4A,B). Given the intricate link between senescence and cell cycle dysregulation, we focused on cell cycle progression. Both PA treatment in vitro (Fig. 4C) and HFD feeding in vivo (Fig. 4D) reduced EdU incorporation in ADSCs, confirming that high fat impairs the ADSC cell cycle.

As EdU incorporation occurs during S-phase and DEGs were enriched at the G1/S transition, we examined key regulators of this checkpoint. CDK4 was significantly downregulated in PA-treated ADSCs (Fig. 4E). Similar CDK4 reduction was observed in adipose tissue from HFD-fed rats, albeit to a lesser extent—consistent with the low abundance of ADSCs in vivo (Fig. 4F). Transcriptome profiling of CDK4-knockdown ADSCs showed enrichment in both cell cycle and senescence pathways (Fig. S4a and b), which was corroborated by pharmacological CDK4 inhibition (Fig. S4c and d). We therefore hypothesized that PA induces senescence through CDK4 downregulation. As expected, CDK4 knockdown impaired ADSC cell cycle progression (Fig. 4G) and induced senescence in both ADSCs and the resulting differentiated adipocytes (hereafter designated as siCDK4-ADSC-MAs) (Fig. 4H). Conversely, CDK4 supplementation rescued PA-induced cell cycle defects and senescence (Figure 4G,H). Although total GLUT4 remained unchanged in siCDK4-ADSC-MAs (Fig. 4I), insulin-stimulated GLUT4 translocation and glucose uptake were severely impaired (Figure 4J,K). These findings demonstrate that NEFAs downregulate CDK4, disrupt cell cycle progression, promote ADSC senescence, and ultimately impair GLUT4 translocation and glucose uptake in ADSC-MAs.

### 3.5 TRIP12-mediated ubiquitination of CDK4 under NEFA stress promotes ADSC senescence

**Figure 5:**
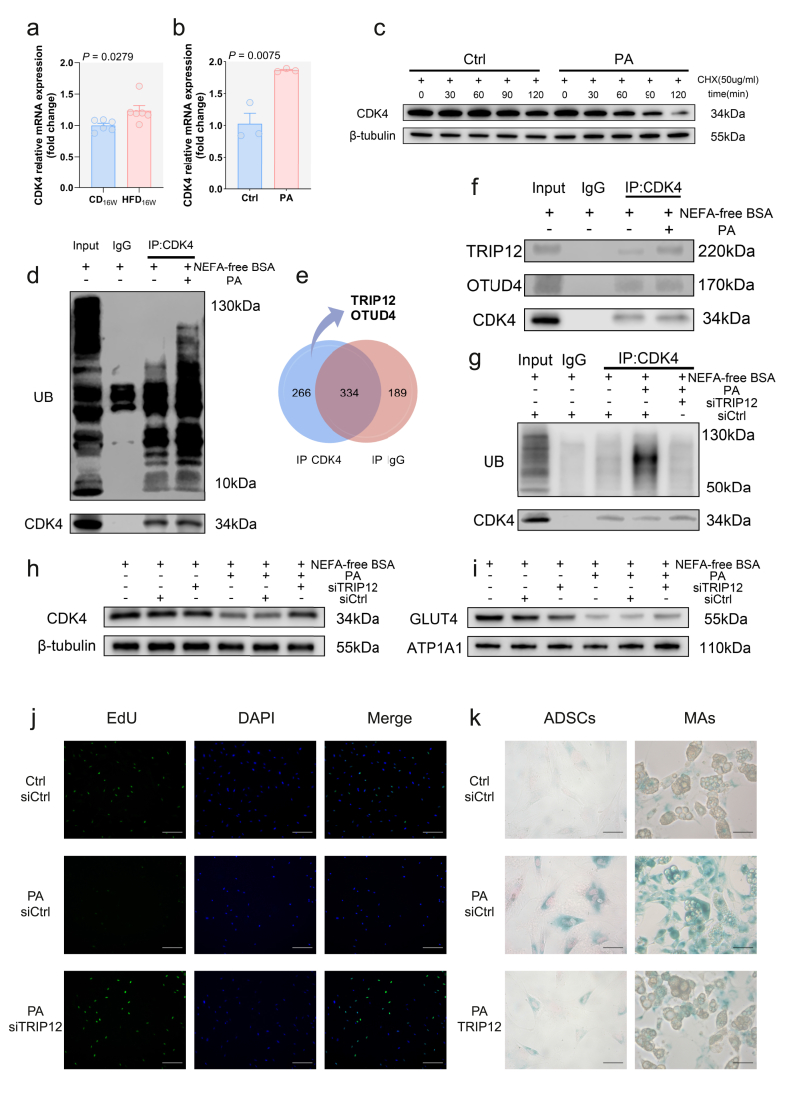
**TRIP12-mediated ubiquitination of CDK4 under NEFA stress promotes ADSC senescencea**, **b** CDK4 mRNA expression in response to high fat stimulation **a** in vivo (n = 6) and **b** in vitro (n = 3), data were analyzed by two-tailed Student's t-test and are presented as mean ± SEM. **c** Protein stability assay. **d** Ubiquitination of CDK4 was detected by WB after Co-IP. **e** Venn diagram of ubiquitination regulator screening and **f** WB validation of the candidate proteins. **g** Ubiquitinated CDK4 detected by WB. **h**, **i** WB images of **h** CDK4 in ADSCs and **i** membrane GLUT4 in corresponding ADSC-MAs. **j** EdU staining of ADSCs, scale bar represents 200um. **k** SA-β-gal staining of ADSCs/ADSC-MAs, scale bar represents 50um.

Following high-fat stimulation, CDK4 protein levels were significantly downregulated in ADSCs (Fig. 4E), while its mRNA levels were paradoxically upregulated both in vitro and in vivo (Figure 5A,B). We therefore assessed CDK4 protein stability and observed a rapid decrease in CDK4 after PA treatment (Fig. 5C), indicating that CDK4 downregulation is primarily mediated through reduced protein stability, with the mRNA increase likely representing a compensatory response. As expected, Co-IP results showed increased ubiquitin binding to CDK4 in PA-treated ADSCs (Fig. 5D), indicating that high fat downregulates CDK4 protein levels by promoting its ubiquitination.

To investigate how high fat promotes CDK4 ubiquitination, we identified two regulatory enzymes: the E3 ubiquitin ligase TRIP12 and the deubiquitinase OTUD4 (Fig. 5E). Both exhibited enhanced binding to CDK4 in PA-treated ADSCs (Fig. 5F). Given their established functions, we hypothesized that TRIP12 plays a major role in this process. Supporting this, PA-treated TRIP12-knockdown ADSCs showed reduced CDK4 ubiquitination (Fig. 5G) and full restoration of CDK4 protein levels (Fig. 5H), demonstrating that TRIP12 is essential for high fat-induced CDK4 downregulation. We next assessed cell cycle progression, senescence, and GLUT4 translocation. TRIP12 knockdown prevented PA-induced impairment of EdU incorporation (Fig. 5J). Interestingly, although SA-β-Gal staining showed reduced senescence in PA + siTRIP12 group compared to the PA + siCtrl group, levels remained higher than in the Ctrl + siCtrl group (Fig. 5K). Insulin-stimulated GLUT4 membrane translocation was only partially restored (Fig. 5I).

We found that both TRIP12 and OTUD4 were upregulated in ADSCs after PA stimulation (Fig. S3h), hinting that high fat may promote extensive protein ubiquitination beyond CDK4, with deubiquitinase upregulation representing a compensatory response.

### 3.6 In an HFD setting, dietary fiber supplementation reverses cellular senescence, hinting the involvement of a gut microbiota-SCFAs-CDK4 axis

**Figure 6:**
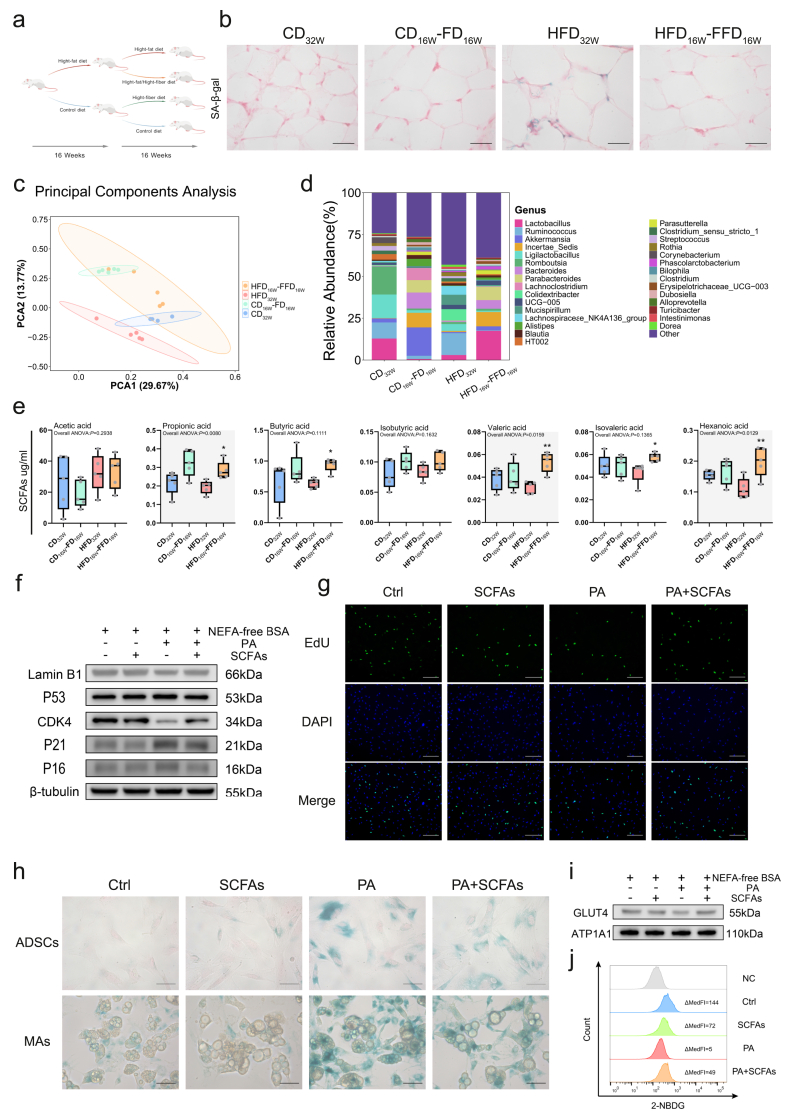
**In an HFD setting, dietary fiber supplementation reverses cellular senescence, hinting the involvement of a gut microbiota-SCFAs-CDK4 axisa** Schematic diagram of the rat feeding protocol. Created with BioGDP.com [51] **b** SA-β-gal staining (Scale bar,50um). **c**, **d** 16S rRNA gene sequencing characterizing **c** microbial beta diversity via PCA and profiling **d** top 30 genera in taxonomic composition. **e** SCFAs concentrations in rat serum. Data are presented as the median ± interquartile range. Whiskers mean minimum and maximum. Data were analyzed by one-way ANOVA and Bonferroni's test was specifically applied to the prespecified comparison between HFD_32W_ and HFD_16W_-FFD_16W_ groups. Gray shading indicates significant difference (*P* < 0.05) of overall one-way ANOVA. ∗∗∗∗*P* < 0.0001; ∗∗∗*P* < 0.001; ∗∗*P* < 0.01; ∗*P* < 0.05. Significance symbols denote HFD_16W_-FFD_16W_ vs. HFD_32W_ comparison. **f** WB images of CDK4 and senescence biomarkers in ADSCs. **g** EdU staining of ADSCs (Scale bar, 200um). **h** SA-β-gal staining of ADSCs/ADSC-MAs (Scale bar, 50um). **i** Membrane GLUT4 in ADSC-MAs differentiated from ADSCs in **f**. **j** Flow cytometric analysis of 2-NBDG uptake in ADSC-MAs under different treatment of CD_16W_ group-derived cells.

Considering the beneficial effects of a high-fiber diet on metabolism, we wondered whether it could alleviate adipose tissue aging induced by an HFD. After 16 weeks on either CD or HFD fed, rats received supplementary dietary fiber in CD and HFD for another 16 weeks, forming the CD_32W_, HFD_32W_, CD_16W_-FD_16W_, HFD_16W_-FFD_16W_ groups (Figure 6A). Although the CD_16W_-FD_16W_ group did not exhibit an improving glucose tolerance compared to CD_32W_, the HFD_16W_-FFD_16W_ group showed significantly better glucose tolerance than HFD_32W_ (Fig. S2c–e). Senescence in adipose tissue was also markedly attenuated under the FFD regimen (Figure 6B,S2f). These results demonstrate that dietary fiber supplementation, even without restricting fat intake, effectively mitigates adipocyte senescence and improves glucose tolerance.

16S sequencing revealed distinct gut microbiota profiles in the CD_32W_, CD_16W_-FD_16W_, and HFD_32W_ groups. The HFD_16W_-FFD_16W_ group exhibited an intermediate composition between CD_16W_-FD_16W_ and CD_32W_ gropus, with high intra-group variability (Figure 6C,D, S2g-i), indicating that fiber supplementation shifts HFD-induced microbiota toward a healthier state resembling control or fiber-enriched patterns.

As expected, we detected increased concentrations of multiple SCFAs in the serum of HFD_16W_-FFD_16W_ rats compared to the HFD_32W_ group (Fig. 6E). Adding SCFAs to PA-treated ADSCs partially restored CDK4 expression (Fig. 6F) and rescued cell cycle arrest (Fig. 6G), yet only moderately attenuated senescence in both ADSCs and adipocytes derived from them (Fig. 6H). While SCFAs supplementation during PA treatment of ADSCs improved insulin-stimulated glucose uptake in ADSC-MAs (Figure 6I,J), it unexpectedly impaired this function in ADSC-MAs derived from ADSCs not pre-treated with PA (Fig. 6J). These findings indicate that SCFAs alone are not universally beneficial but predominantly exert positive effects under high-fat conditions.

## 4 Discussion

Our study characterizes a sequence of events in which HFD induces senescence in both ADSCs and resulting adipocytes, involving disruption of the ADSC cell cycle and coinciding with the development of impaired glucose tolerance. These findings not only suggest immediate therapeutic targets for glucose intolerance but also open avenues for potentially reversing adipose tissue senescence. Moreover, we demonstrate that a high-fiber diet counteracts HFD-induced senescence in both ADSCs and MAs and restores glucose uptake capacity in MAs. We speculate that these beneficial effects involve the gut microbiota–SCFAs–CDK4 pathway. This provides an explanatory framework for the benefits of fiber supplementation in alleviating HFD-induced adipose tissue senescence and offers a practical dietary intervention for improving glucose tolerance, which may be more feasible than strict fat restriction.

Cellular senescence is a natural process driven by complex molecular mechanisms that impair normal physiological function [8]. While HFD-induced adipose tissue senescence is often attributed to obesity-related chronic inflammation [4,28,33], our study shows that it is developed independently of obesity. We identified a previously underreported phenomenon: NEFAs directly promote senescence in ADSCs. These senescent ADSCs differentiate into dysfunctional MAs. Although reduced GLUT4 expression and impaired membrane translocation are known features of senescent adipose tissue [11,34], HFD-induced senescence specifically disrupts insulin-stimulated GLUT4 translocation without reducing total protein levels (Figure 3G,I). This is associated with insulin-resistant glucose dysregulation (Figure 3E,F). Furthermore, senescent adipose tissue exhibits a low-grade chronic inflammatory state, consistent with the activation of multiple SASP factors—which serve as both senescence markers and inflammatory mediators [35].

Senescence is typically accompanied by a progressive decline in the differentiation capacity of stem cells [36,37]. In our study, however, ADSCs induced into senescence by high-fat stimulation—both in vivo and in vitro—retained the ability to differentiate into MAs containing prominent lipid droplets (Figure 3C,D), although these cells exhibited significant impairment in GLUT4 membrane translocation and compromised glucose uptake capability (Figure 3.e-j). This dissociation between differentiation capacity and metabolic function suggests that high-fat stimulation may induce a distinct senescent state in ADSCs, characterized by compromised metabolic function while temporarily preserving differentiation capability [38]. This process ultimately drives the formation of functionally impaired MAs, revealing a cell-autonomous aspect of how high-fat stimulation affects adipose tissue function. In the in vivo microenvironment, senescent ADSCs further remodel the local environment by secreting SASP, including various inflammatory factors and chemokines [39,40]. These factors not only induce insulin resistance in surrounding healthy adipocytes in a paracrine manner but also exacerbate systemic metabolic dysfunction by establishing a chronic inflammatory state [41,42]. Therefore, the observed glucose intolerance may result from the combined effects of cell-autonomous functional defects and microenvironmental dysregulation [43].

Cellular senescence can be defined as an irreversible arrest of the cell cycle [7,8], which led us to focus on cell cycle regulation. Although MAs exhibit an incomplete cell cycle, it does not support effective replication [27]. While some studies suggest this incomplete cycle is linked to adipocyte senescence [27,28], we propose it may simply be a non-functional remnant of incomplete differentiation. The investigation was therefore focused on ADSCs, and our experimental results strongly support this direction (Figure 3C,D, S3a). While extensive research has focused on utilizing ADSCs to alleviate aging in other tissues [44,45], little attention has been paid to the senescence of ADSCs themselves. Particularly, the intrinsic link between ADSC senescence and their impaired cell cycle progression remains poorly understood. CDK4, which we identified as central to HFD-induced senescence, plays a key role in the G1/S transition [46]. Although studies on CDK4 in adipose tissue are limited, its role is well-established in other contexts. For example, the CDK4 inhibitor Palbociclib treats breast cancer by inducing cell cycle arrest and senescence [47,48]. We demonstrate that CDK4 is equally critical in regulating the cell cycle and senescence in ADSCs. Earlier studies indicated that CDK4 is essential for adipogenesis, with its deficiency impairing adipocyte differentiation in embryonic fibroblasts [49]. While adipogenic capacity was largely preserved in our model, considering that stemness declines with cellular senescence, our findings can be reconciled with this established role.

The molecular mechanism underlying NEFA-induced cell cycle disruption and senescence in ADSCs is attributed to CDK4 ubiquitination and degradation. Prior to this study, the ubiquitination-dependent degradation of CDK4 had not been fully elucidated. We identified TRIP12 as the key E3 ubiquitin ligase catalyzing this process. Furthermore, our data suggest that OTUD4, a deubiquitinase known to regulate CDK1 [50], may also be involved in controlling CDK4 ubiquitination. However, its precise regulatory mechanism requires further investigation (Fig. 5F).

High-fiber diets are well-established to improve glucose tolerance [19,21]. Our results emphasize that even under continued high fat intake, fiber supplementation alone is sufficient to reverse cellular senescence in adipose tissue and improve glucose tolerance in animal models (Figure 6B S2c-e). The most immediate effect of dietary fiber relates to modulation of the gut microbiota [20]. Consistent with previous studies [22], high-fiber feeding increased the abundance of Bacteroides and reduced Ruminococcus (Fig. 6D), promoting the production of SCFAs (Fig. 6E). Beyond the established links between the fiber–microbiota–SCFAs axis and diverse systemic effects [21,22], our study specifically identifies a significant association with the reversal of both senescence and metabolic dysfunction.

We acknowledge several limitations in this study. Adipose samples were obtained from patients with early-stage colorectal cancer for practical accessibility. While this sampling strategy may raise concerns about generalizability, there is currently little evidence that localized colorectal cancer alters adipose tissue biology. Furthermore, because cell-based models cannot fully replicate the chronic lipid exposure seen in vivo, we corroborated our key findings in animal models. Additionally, we did not employ adipogenic markers to achieve rigorous quantification of adipogenesis in ADSCs. Although our cells, including the senescent populations, did not exhibit impaired adipogenesis under microscopic examination, we cannot rule out the potential influence of subtle differences in differentiation capacity. Moreover, given the inherent heterogeneity in adipogenic differentiation efficiency, the co-existence of ADSCs at varying differentiation stages may exert paracrine effects on normal MAs—a possibility that remains to be confirmed but cannot be disregarded. Finally, while this study focuses on cell-intrinsic changes, future investigations should incorporate the roles of intercellular communication and microenvironmental influences in metabolic regulation.

Research on reversing senescence is both profoundly significant and highly practical. As a key metabolic regulator, the rejuvenation of adipose tissue could offer novel clinical insights for treating metabolic diseases. Although this study has certain limitations, it proposes that targeting adipose dysfunction through a previously underexplored avenue—the cell cycle—may reveal valuable metabolic pathways, underlying processes, and molecular targets.

## CRediT authorship contribution statement

**Zheng Ge:** Writing – original draft, Visualization, Investigation, Formal analysis, Data curation, Conceptualization. **Zitian Liu:** Writing – review & editing, Methodology, Investigation. **Shuohui Dong:** Writing – review & editing, Methodology, Conceptualization. **Xiang Zhao:** Investigation. **Guangwei Yang:** Writing – review & editing. **Ao Yu:** Investigation. **Wei Guo:** Writing – review & editing, Validation. **Xiang Zhang:** Writing – review & editing, Validation. **Qunzheng Wu:** Investigation. **Kexin Wang:** Writing – review & editing, Supervision, Resources, Project administration, Funding acquisition.

## Funding

This work was supported by the 10.13039/501100001809National Natural Science Foundation of China (General Program), No. 82070852 and No. 82270901.

## Declaration of competing interest

The authors declare no conflict of interest.

## Data Availability

Data will be made available on request.
