# Supplementary material for: High-fat diet induces senescence in ADSCs via CDK4 ubiquitination-mediated cell cycle disruption, contributing to impaired glucose tolerance
Source: Mol Metab. 2025 Nov 29;103:102293. doi: 10.1016/j.molmet.2025.102293 (PMC12743421; doi:10.1016/j.molmet.2025.102293)
Supplement: Multimedia component 1 [file mmc1.pdf]

Supplementary Material

High-Fat Diet Induces Senescence in ADSCs via CDK4 Ubiquitination-Mediated Cell Cycle Disruption, Contributing to Impaired Glucose Tolerance

Zheng Ge, Zitian Liu, Shuohui Dong, Xiang Zhao, Guangwei Yang, Ao Yu, Wei Guo, Xiang Zhang, Qunzheng Wu, Kexin Wang*

Table S1 Clinical data of the human subjects in the fat intake score and fasting blood glucose analysis (n=48)

Patient characteristics	Category	Frequency (number)
**Age (years) [a]**	<40	2.08% (1)
	40-49	10.42% (5)
	50-59	31.25% (15)
	60-69	41.67% (20)
	≥70	14.58% (7)
**Sex**	Male	47.92% (23)
	Female	52.08% (25)
**BMI (kg/m²) [b]**	<18.5	2.08% (1)
	18.5-24	54.17% (26)
	>24	43.75% (21)
**TNM stage**	T1N0M0	4.17% (2)
	T2N0M0	31.25% (15)
	T3N0M0	64.58% (31)
	Others	0.00% (0)

[a] The age ranges were 35–75 years

[b] The BMI ranges were 17.5–36.3 kg/m²

Table S2 Clinical data of the human subjects in the fat intake score and insulin-related analyses (n=41)

Patient characteristics	Category	Frequency (number)
**Age (years)** [a]	<40	2.44% (1)
	40-49	9.76% (4)
	50-59	31.71% (13)
	60-69	39.02% (16)
	≥70	17.07% (7)
**Sex**	Male	48.78% (20)
	Female	51.22% (21)
**BMI (kg/m^2)** [b]	<18.5	2.44% (1)
	18.5-24	51.22% (21)
	>24	46.34% (19)
**TNM stage**	T1N0M0	4.88% (2)
	T2N0M0	34.15% (14)
	T3N0M0	60.98% (25)
	Others	0.00% (0)

[a] The age ranges were 35–75 years

[b] The BMI ranges were 17.5–36.3 kg/m^2

Table S3 Clinical data of the human subjects in the RNA sequencing cohort (n=16)

Patient characteristics	Category	Frequency (number)
**Cohort 1. lower-fat-intake group (n=8)**		
**Age (years)** [a]	<40	0.00% (0)
	40-49	12.50% (1)
	50-59	37.50% (3)
	60-69	50.00% (4)
	≥70	0.00% (0)
**Sex**	Male	62.50% (5)
	Female	37.50% (3)
**BMI (kg/m²)** [b]	<18.5	12.50% (1)
	18.5-24	62.50% (5)
	>24	25.00% (2)
**TNM stage**	T1N0M0	0.00% (0)
	T2N0M0	37.50% (3)
	T3N0M0	62.50% (5)
	Others	0.00% (0)
**Cohort 2. Higher-fat-intake group (n=8)**		
**Age (years)** [a]	<40	0.00% (0)
	40-49	12.50% (1)
	50-59	50.00% (4)
	60-69	25.00% (2)
	≥70	12.50% (1)
**Sex**	Male	62.50% (5)
	Female	37.50% (3)
**BMI (kg/m²)** [b]	<18.5	0.00% (0)
	18.5-24	12.50% (1)
	>24	87.50% (7)
**TNM stage**	T1N0M0	0.00% (0)
	T2N0M0	25.00% (2)
	T3N0M0	75.00% (6)
	Others	0.00% (0)

[a] The age ranges were 42–67 years for Cohort 1 and 49–71 years for Cohort 2.

[b] The BMI ranges were 17.5–26.4 kg/m² for Cohort 1 and 22.8–36.3 kg/m² for Cohort 2.

Table S4 Dietary Fat Intake Assessment Questionnaire

Please recall your dietary habits over the past year (including breakfast, lunch, dinner, and eating out), and select the most appropriate frequency option for each listed food or beverage item.

		Less than 1 per month	2-3 per month	1-2 per week	3-4 per week	5+ per week
1	Mince, beef or pork, for example, in hamburgers, Chinese meat burger					
2	Beef or pork such as steak, ribs, roasts or in sandwiches					
3	Fried chicken or chicken burgers					
4	Sausages					
5	Bacon					
6	Chinese sesame paste, Salad dressings (not low fat)					
7	Margarine, butter or oil in cooking					
8	Eggs (not egg whites alone)					
9	Pizza, Meat Pie					
10	Chili oil sauce, Chili black bean sauce (not low fat)					
11	French fries, fried potatoes					
12	Corn chips, potato chips, popcorn with butter					
13	Doughnuts, pastries, croissants					
14	Cakes, cookies					
15	Ice cream (not sorbet or low fat)					
16	Chocolate					
17	Spreads incl. peanut butter, jam					
18	Pancakes, fried dough cake, fried dough stick					
19	Milk (full fat only). Include milk drunk by itself or in cappuccinos, milkshakes, hot chocolates etc					
20	In the past year, how many times have you eaten food from a takeaway or fast food restaurant for example McDonalds, KFC					

Table S5 Antibodies used in this study

Antibodies for Immunofluorescence				
Antibody Type	Protein Target	Manufacturer	Catalog Number	Dilution
primary antibody	CD105	Proteintech	10862-1-AP	1:100
primary antibody	CD29	ABclonal	A23497	1:100
secondary antibody	/	Proteintech	SA00013-4	1:100
Antibodies for Immunohistochemistry				
Antibody Type	Protein Target	Manufacturer	Catalog Number	Dilution
primary antibody	CD105	Proteintech	10862-1-AP	1:1000
primary antibody	CD29	ABclonal	A23497	1:1000
secondary antibody	/	ZSGB-BIO	PV9000	Ready-to-use
Antibodies for Western Blot				
Antibody Type	Protein Target	Manufacturer	Catalog Number	Dilution
primary antibody	β-tubulin	Proteintech	80713-1-RR	1:5000
primary antibody	Lamin B1	Selleck	F0523	1:1000
primary antibody	P53	Proteintech	60283-2-Ig	1:5000
primary antibody	P21	PTMAL	PTM-7125	1:1000
primary antibody	P16	ABclonal	A23882	1:500
primary antibody	GLUT1	ABclonal	A6982	1:1000
primary antibody	GLUT4	ABclonal	A7637	1:500
primary antibody	ATP1A1	Selleck	F2192	1:10000
primary antibody	CDK4	Abcam	AB226474	1:2000
primary antibody	Ubiquitin	Cell Signaling Technology	#3936	1:1000
primary antibody	TRIP12	ABclonal	A9958	1:1000
primary antibody	OTUD4	ABclonal	A15229	1:1000
secondary antibody	/	Proteintech	SA00001-1	1:5000
secondary antibody	/	Proteintech	SA00001-2	1:5000
Antibodies for Immunoprecipitation				
Capture Antibody	CDK4	Abcam	AB226474	10 μg/mg of lysate

Table S6 Primers used in this study

Gene name	Forward sequence (5'to 3')	Reverse sequence (5'to 3')
Human primers		
IL1β	GCACCTGTACGATCACTGAACTG	CACTTGTTGCTCCATATCCTGTCC
PAI1	GGCTGGTGCTGGTGAATGC	AGTGCTGCCGTCTGATTTGTG
TIMP1	CCTGTTGTTGCTGTGGCTGATAG	CTGATGACGAGGTCGGAATTGC
MIP2	ATCCAAAGTGTGAAGGTGAAGTCC	AGCTTTCTGCCCATTCTTGAGTG
CXCL1	CCGAAGTCATAGCCACACTCAAG	GTTGGATTTGTCACTGTTCAGCATC
MMP14	TCCATCAACACTGCCTACGAGAG	ACGCCTCATCAAACACCCAATG
CTGF	GTTACCAATGACAACGCCTCCTG	TTGCCCTTCTTAATGTTCTCTTCCAG
IL6	GGTGTTGCCTGCTGCCTTCC	TGAGATGCCGTCGAGGATGTACC
MCP1	GACCATTGTGGCCAAGGAGA	TTGGGTTTGCTTGTCCAGGT
IL8	CTCTTGGCAGCCTTCCTGATTTC	GGGTGGAAAGGTTTGGAGTATGTC
IP10	TGCCATTCTGATTTGCTGCC	GCTGATGCAGGTACAGCGTA
MMP19	CTGACATCCGCCTCTCCTTCC	GCTCTGTCTCTTCTTCTTCCTCATC
ADAMTS4	CGCTTTGCTTCACTGAGTAGATTTG	CTGCTGCTGCCATCACTGTTAG
PPIA	TCTGCACTGCCAAGACTGAG	TCGAGTTGTCCACAGTCAGC
Rat primers		
TNF-α	CTGTGCCTCAGCCTCTTCTC	ACTGATGAGAGGGAGCCCAT
IL1β	CACCTCTCAAGCAGAGCACA	CGGGTTCCATGGTGAAGTCA
CXCL1	GGCAGGGATTCACTTCAAGAACATC	TGTGGCTATGACTTCGGTTTGGG
MIP2	TGTACTGGTCCTGCTCCTCCTG	TCACCGTCAAGCTCTGGATGTTC
CTGF	CGGGAAATGCTGTGAGGAGT	ACAGGTCTTAGAACAGGCGC
MMP14	TGAGGAGGAGACGGAGGTGATC	CAGTACCAGGAGCAGCAGCAG
IL6	CTTCCAGCCAGTTGCCTTCTTG	TGGTCTGTTGTGGGTGGTATCC
MMP19	CCTGTTTACGCTGGCTACCA	CCTCCTCTTGCCATAGAGCG
IP10	GCAAGTCTATCCTGTCCGCA	AGACCTTCTTTGGCTCACCG
PAI1	CTACCACGGCGAAACCCTCAG	GGTCATGTTGCTCTTCCATTGTCTG
TIMP1	CATGGAGAGCCTCTGTGGATATGTC	ATTATGCCAGGGAACCAGGAAGC
MCP1	CCACTCACCTGCTGCTACTC	ACCTGCTGCTGGTGATTCTC
CDK4	ATGTCTGTGCTACTTCCCGAACTG	TGCTTTGTCCAGGTATGTCCGTAG
PPIA	GCACTGGTGGCAAGTCCATCTAC	TGCTCATGCCTTCTTTCACCTTCC

Table S7 siRNA duplexes used in this study

siRNA Target	Sense	Antisense
CDK4(rat)	5’-CUGACCUUUAAUCCACUUATT-3’	5’-UAAGUGGAUUAAAGGUCAGTT-3’
TRIP12(rat)	5’-GGUUCUGGGUCCGAAUCAATT-3’	5’-UUGAUUCGGACCCAGAACCTT-3’
Non-targeting Ctrl	5’-UUCUCCGAACGUGUCACGUTT-3’	5’-ACGUGACACGUUCGGAGAATT-3’

Table S8 Sequence of the CDK4 plasmid

Gene name	Sequence
CDK4(rat)	ATGGCTACCACTCGATATGAACCCGTGGCTGAAATTGGTGTCG<br>GTGCCTATGGGACGGTGTACAAAGCCCGAGATCCCCACAGTG<br>GCCACTTTGTGGCTCTCAAGAGTGTGAGAGTTCCTAATGGAG<br>GAGCAGCTGGAGGGGGCCTTCCCGTCAGCACAGTTCGTGAG<br>GTGGCCTTGTTAAGAAGGCTGGAGGCCTTTGAACATCCCAAT<br>GTTGTACGGCTGATGGATGTCTGTGCTACTTCCCGAACTGATC<br>GGGACATCAAGGTCACCTTAGTGTTTGAGCATATAGACCAGGA<br>CCTACGGACATACCTGGACAAAGCACCTCCGCCGGGCTTGCC<br>TGTTGAGACCATTAAGGATCTGATGCGCCAGTTTCTAAGCGGC<br>CTAGATTTCCTTCATGCAAACTGCATTGTTCACCGGGACCTGA<br>AGCCAGAGAACATTCTAGTGACAAGTAATGGGACAGTTAAGCT<br>GGCCGACTTTGGCCTAGCCAGAATCTACAGCTACCAGATGGC<br>CCTCACGCCTGTGGTTGTTACGCTCTGGTACCGGGCTCCTGA<br>AGTTCTTCTGCAGTCTACATATGCAACGCCTGTGGATATGTGG<br>AGTGTTGGCTGTATCTTCGCAGAGATGTTTCGCCGGAAGCCTC<br>TCTTCTGTGGGAACTCTGAGGCTGACCAGCTGGGCAAAATCT<br>TTGATCTCATTGGATTGCCTCCAGAAGACGACTGGCCTCGAGA<br>GGTCTCTCTTCCTCGAGGAGCCTTTTCCCCCAGAGGACCTCG<br>GCCAGTGCAGTCAGTGGTGCCGGAGATGGAGGAATCTGGAG<br>CGCAGTTGCTGCTGGAAATGCTGACCTTTAATCCACTTAAGCG<br>AATCTCTGCCTTCCGAGCCCTGCAGCACTCTTACCTGCACAAG<br>GAGGAAAGTGACCCGGAGTGA

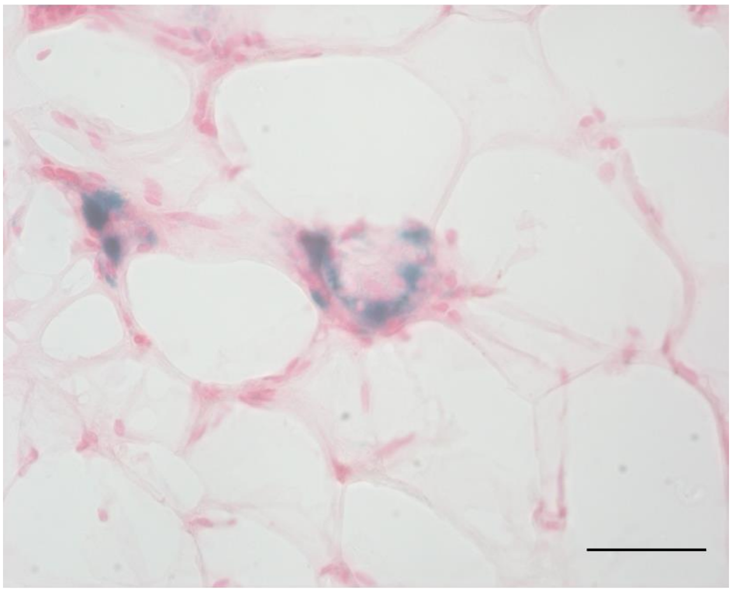

Figure S1 Representative histological section from human volunteer samples
SA-β-gal staining reveals senescent cells within the SVF of subcutaneous adipose tissue from human volunteers with high-fat diet consumption. Scale bar, 50 μm.

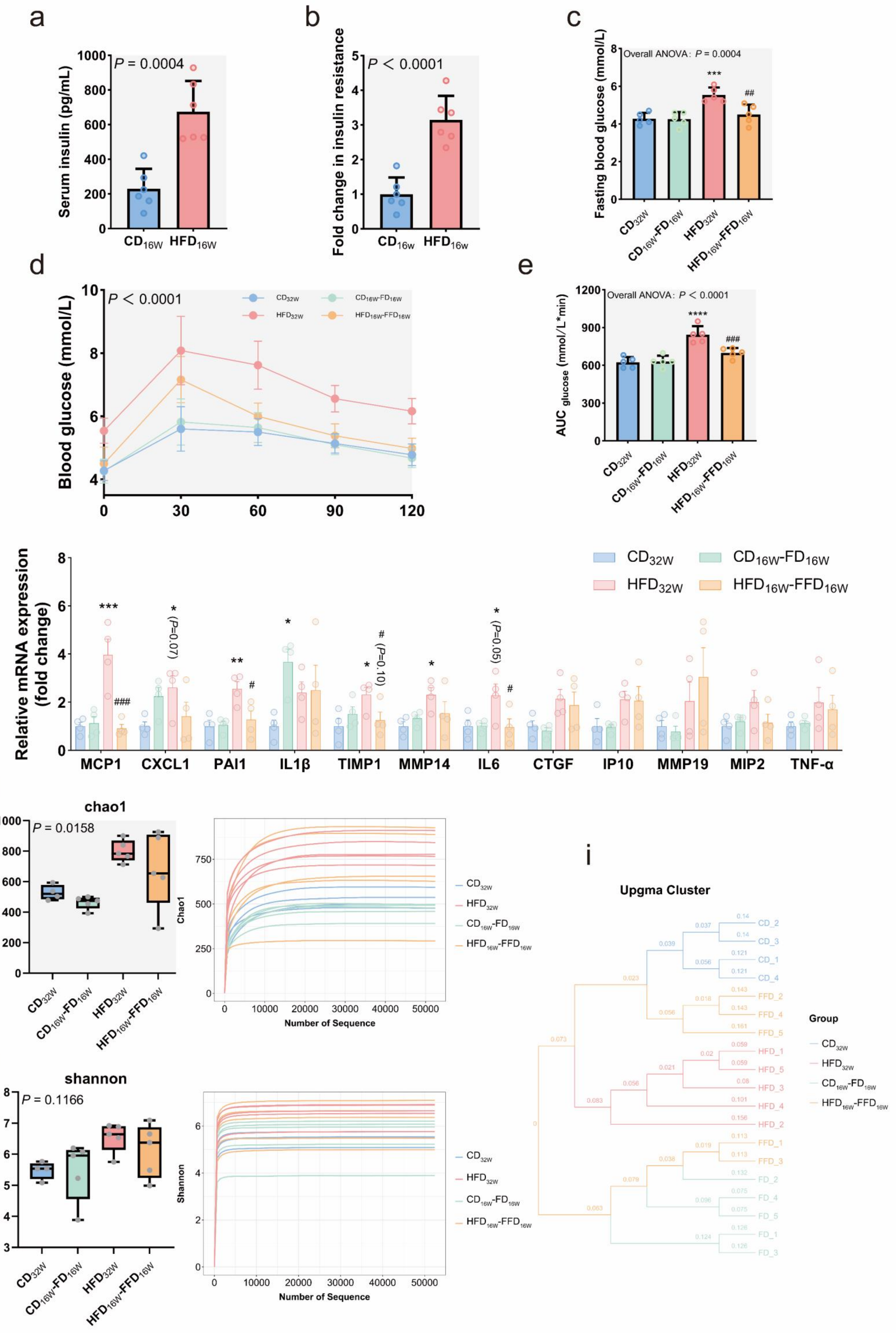
a
P = 0.0004
Serum insulin (pg/mL)
CD16W HFD16W
b
P < 0.0001
Fold change in insulin resistance
CD16w HFD16w
c
Overall ANOVA: P = 0.0004
Fasting blood glucose (mmol/L)
CD32W CD16W-FD16W HFD32W HFD16W-FFD16W
d
P < 0.0001
Blood glucose (mmol/L)
CD32W CD16W-FD16W HFD32W HFD16W-FFD16W
e
Overall ANOVA: P < 0.0001
AUC glucose (mmol/L*min)
f
Relative mRNA expression (fold change)
(P=0.07) (P=0.10) (P=0.05)
MCP1 CXCL1 PAI1 IL1β TIMP1 MMP14 IL6 CTGF IP10 MMP19 MIP2 TNF-α
g
chao1
P = 0.0158
Chao1
Number of Sequence
h
shannon
P = 0.1166
Shannon
i
Upgma Cluster
Group
CD_2 CD_3 CD_1 CD_4 FFD_2 FFD_4 FFD_5 HFD_1 HFD_5 HFD_3 HFD_4 HFD_2 FFD_1 FFD_3 FD_2 FD_4 FD_5 FD_1 FD_3

Figure S2 Experimental data of rat models

a Serum insulin levels. **b** A relative measure of insulin sensitivity, expressed as the ratio of the glucose-insulin product in the experimental group to the baseline level of the control group. **c** Fasting blood glucose levels. **d** Blood glucose levels during the OGTT. **e** The corresponding AUC for the OGTT. **f** mRNA levels of SASP. **g** Community richness assessed by the Chao1 index (boxplot) with its rarefaction curve. **h** Community diversity assessed by the Shannon index (boxplot) with its rarefaction curve. **i** UPGMA clustering dendrogram based on Weighted UniFrac distance. Data in **a**, **b** were analyzed by Student's t-test. Data in **c**, **e**, **f** were analyzed by one-way ANOVA followed by Bonferroni's post-hoc test for specific comparisons: CD_{16W}-FD_{16W} vs CD_{32W}, HFD_{32W} vs CD_{32W}, and HFD_{16W}-FFD_{16W} vs HFD_{32W} for **c**, **e**. HFD_{32W} vs CD_{32W} and HFD_{16W}-FFD_{16W} vs HFD_{32W} for **f**. Data in **d** were analyzed by two-way ANOVA. Data in **g**, **h** were analyzed by the Kruskal-Wallis test. Significant differences are indicated by gray shading in **a**-**e**, **g**, **h** and by asterisks (*, vs CD_{32W}) and hashes (#, vs HFD_{32W}) in **c**, **e**, **f**. Data are presented as mean ± SD in **a**-**e** or mean ± SEM in **f**. Sample sizes are n=6 for **a**, **b**; n=5 for all groups in **c**-**e**; n=4 for all groups in **f**; n=4 for CD_{32W}, n=5 for other groups in **g**-**i**.

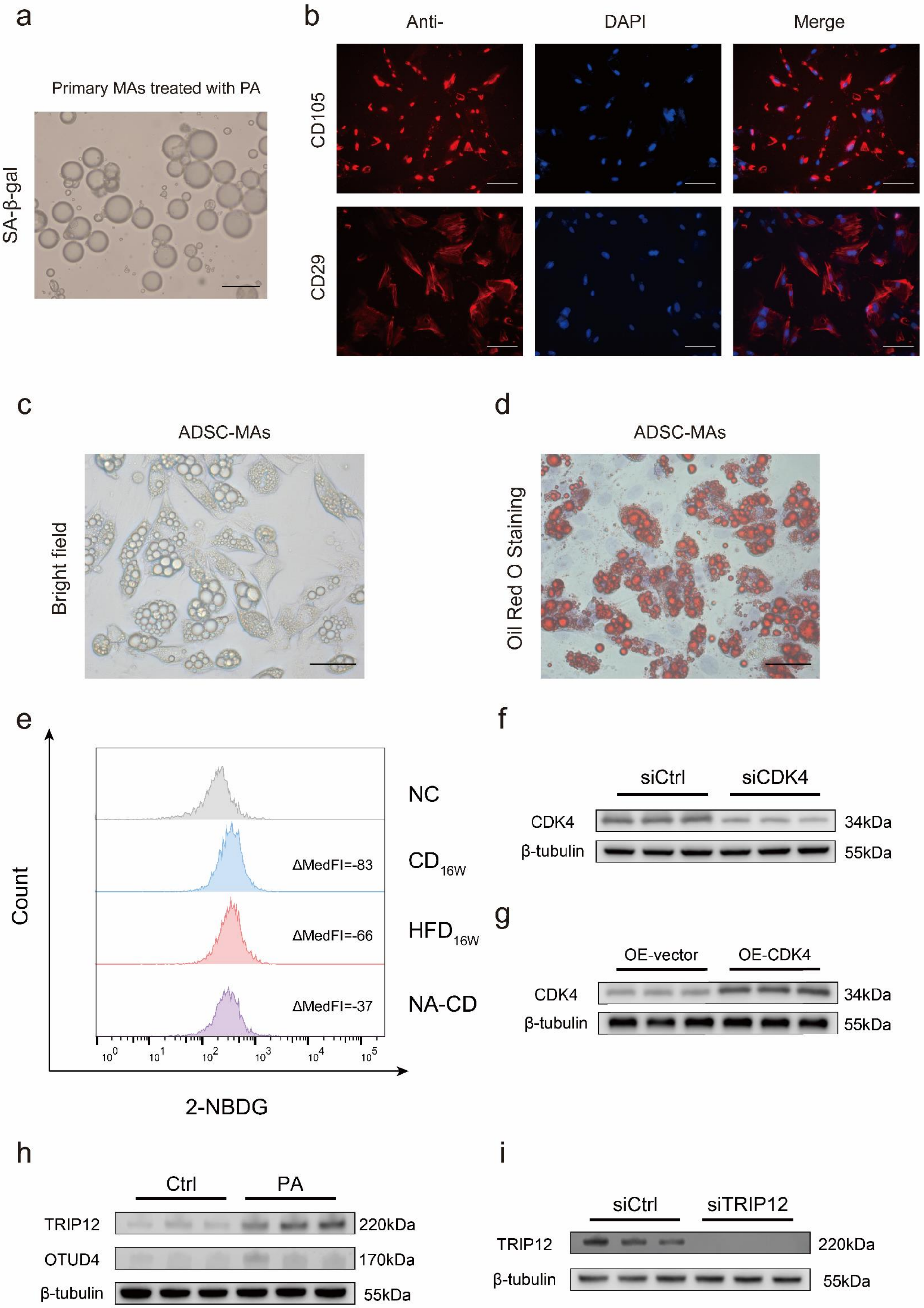
a
Primary MAs treated with PA
SA-β-gal
b
Anti-
DAPI
Merge
CD105
CD29
c
ADSC-MAs
Bright field
d
ADSC-MAs
Oil Red O Staining
e
NC
ΔMedFI=-83
CD16W
ΔMedFI=-66
HFD16W
ΔMedFI=-37
NA-CD
Count
2-NBDG
f
siCtrl
siCDK4
CDK4
β-tubulin
34kDa
55kDa
g
OE-vector
OE-CDK4
CDK4
β-tubulin
34kDa
55kDa
h
Ctrl
PA
TRIP12
OTUD4
β-tubulin
220kDa
170kDa
55kDa
i
siCtrl
siTRIP12
TRIP12
β-tubulin
220kDa
55kDa

Figure S3 Experimental data of cell models

a Representative images of SA-β-gal staining in primary MA isolated from the CD_{16W} group and followed by in vitro PA stimulation. The absence of blue precipitate indicates that no significant cellular senescence was induced by this high-concentration fatty acid environment. **b** Immunofluorescence images confirming the identity of isolated ADSCs, showing positive expression of classic surface markers CD105 and CD29. **c** Morphological appearance of ADSC-MA. **d** Oil Red O staining of ADSC-MAs demonstrating the adipogenic differentiation potential of the isolated ADSCs. **e** Flow cytometric analysis of ADSC-MAs without insulin treatment, showing no significant glucose uptake across all groups. **f**-**i** Western blot images (representative of three independent biological replicates) showing: **f** the knockdown efficiency of siCDK4 oligonucleotides; **g** the overexpression efficiency of CDK4; **h** protein expression levels of TRIP12 and OTUD4 in response to PA stimulation; and **i** the knockdown efficiency of siTRIP12.

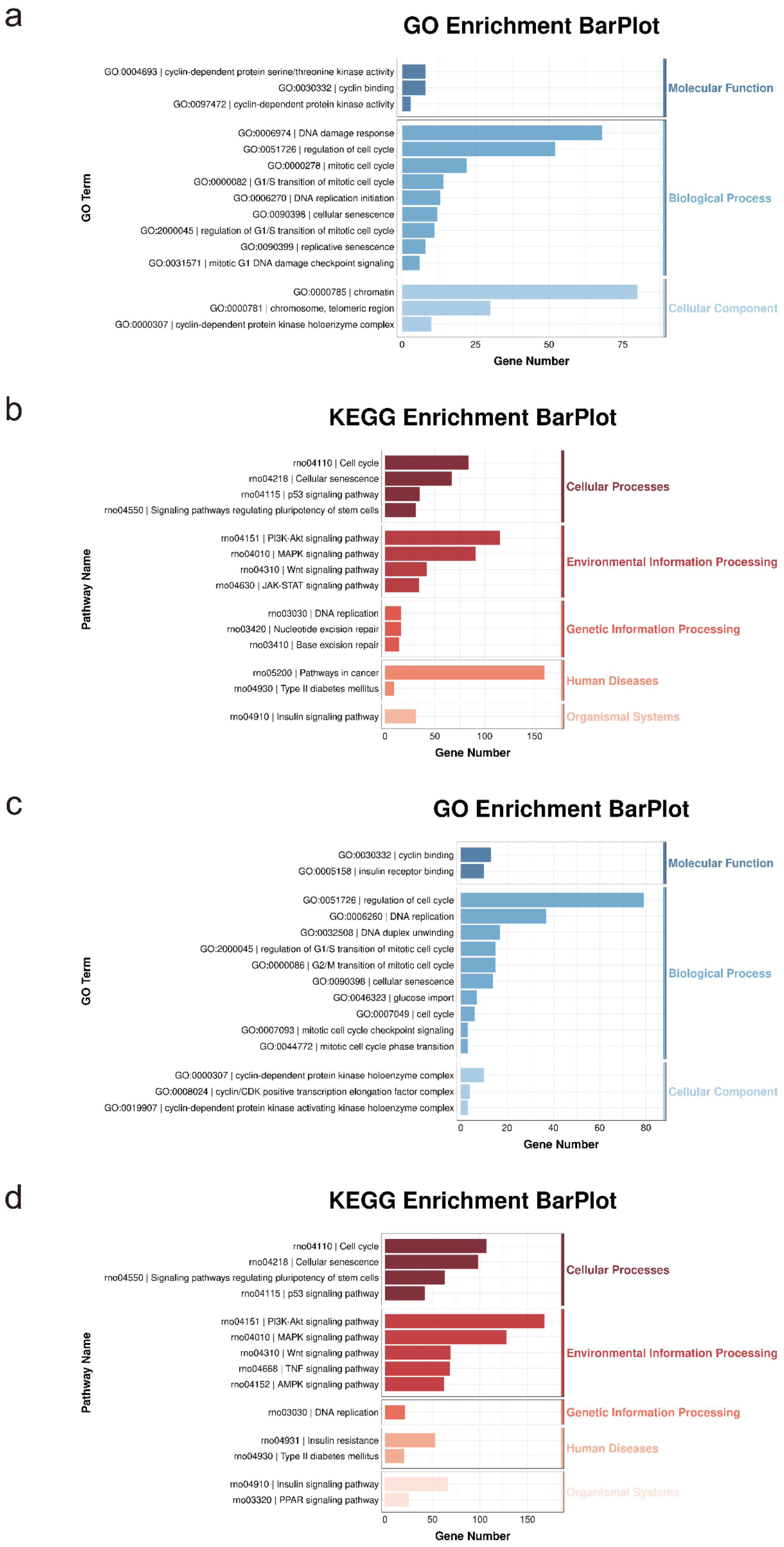

a
GO Enrichment BarPlot
GO:0004693 | cyclin-dependent protein serine/threonine kinase activity
GO:0030332 | cyclin binding
GO:0097472 | cyclin-dependent protein kinase activity
GO:0006974 | DNA damage response
GO:0051726 | regulation of cell cycle
GO:0000278 | mitotic cell cycle
GO:0000082 | G1/S transition of mitotic cell cycle
GO:0006270 | DNA replication initiation
GO:0090398 | cellular senescence
GO:2000045 | regulation of G1/S transition of mitotic cell cycle
GO:0090399 | replicative senescence
GO:0031571 | mitotic G1 DNA damage checkpoint signaling
GO:0000785 | chromatin
GO:0000781 | chromosome, telomeric region
GO:0000307 | cyclin-dependent protein kinase holoenzyme complex
Molecular Function
Biological Process
Cellular Component
GO Term
0
25
50
75
Gene Number
b
KEGG Enrichment BarPlot
rno04110 | Cell cycle
rno04218 | Cellular senescence
rno04115 | p53 signaling pathway
rno04550 | Signaling pathways regulating pluripotency of stem cells
rno04151 | PI3K-Akt signaling pathway
rno04010 | MAPK signaling pathway
rno04310 | Wnt signaling pathway
rno04630 | JAK-STAT signaling pathway
rno03030 | DNA replication
rno03420 | Nucleotide excision repair
rno03410 | Base excision repair
rno05200 | Pathways in cancer
rno04930 | Type II diabetes mellitus
rno04910 | Insulin signaling pathway
Cellular Processes
Environmental Information Processing
Genetic Information Processing
Human Diseases
Organismal Systems
Pathway Name
0
50
100
150
Gene Number
c
GO Enrichment BarPlot
GO:0030332 | cyclin binding
GO:0005158 | insulin receptor binding
GO:0051726 | regulation of cell cycle
GO:0006260 | DNA replication
GO:0032508 | DNA duplex unwinding
GO:2000045 | regulation of G1/S transition of mitotic cell cycle
GO:0000086 | G2/M transition of mitotic cell cycle
GO:0090398 | cellular senescence
GO:0046323 | glucose import
GO:0007049 | cell cycle
GO:0007093 | mitotic cell cycle checkpoint signaling
GO:0044772 | mitotic cell cycle phase transition
GO:0000307 | cyclin-dependent protein kinase holoenzyme complex
GO:0008024 | cyclin/CDK positive transcription elongation factor complex
GO:0019907 | cyclin-dependent protein kinase activating kinase holoenzyme complex
Molecular Function
Biological Process
Cellular Component
GO Term
0
20
40
60
80
Gene Number
d
KEGG Enrichment BarPlot
rno04110 | Cell cycle
rno04218 | Cellular senescence
rno04550 | Signaling pathways regulating pluripotency of stem cells
rno04115 | p53 signaling pathway
rno04151 | PI3K-Akt signaling pathway
rno04010 | MAPK signaling pathway
rno04310 | Wnt signaling pathway
rno04668 | TNF signaling pathway
rno04152 | AMPK signaling pathway
rno03030 | DNA replication
rno04931 | Insulin resistance
rno04930 | Type II diabetes mellitus
rno04910 | Insulin signaling pathway
rno03320 | PPAR signaling pathway
Cellular Processes
Environmental Information Processing
Genetic Information Processing
Human Diseases
Organismal Systems
Pathway Name
0
50
100
150
Gene Number

Figure S4 GO and KEGG enrichment analysis of cell models
a GO and **b** KEGG enrichment analysis of DEGs between siCDK4 and Control groups.
c GO and **d** KEGG enrichment analysis of DEGs between Palbociclib (inhibitor of CDK4) treated and Control groups.
